# Development and Feasibility Testing of Internet-Delivered Acceptance and Commitment Therapy for Severe Health Anxiety: Pilot Study

**DOI:** 10.2196/mental.9198

**Published:** 2018-04-06

**Authors:** Ditte Hoffmann, Charlotte Ulrikka Rask, Erik Hedman-Lagerlöf, Brjánn Ljótsson, Lisbeth Frostholm

**Affiliations:** ^1^ The Research Clinic for Functional Disorders and Psychosomatics Aarhus University Hospital Aarhus Denmark; ^2^ Child and Adolescent Psychiatric Centre Aarhus University Hospital Aarhus Denmark; ^3^ Department of Clinical Neuroscience Karolinska Institute Stockholm Sweden; ^4^ Centre for Psychiatry Research Karolinska Institute Stockholm Sweden

**Keywords:** health anxiety, illness anxiety disorder, hypochondriasis, internet intervention, feasibility, acceptance and commitment therapy, behavior therapy

## Abstract

**Background:**

Severe health anxiety (hypochondriasis), or illness anxiety disorder according to the Diagnostic and Statistical Manual of Mental Disorders, 5th edition, is characterized by preoccupation with fear of suffering from a serious illness in spite of medical reassurance. It is a debilitating, prevalent disorder associated with increased health care utilization. Still, there is a lack of easily accessible specialized treatment for severe health anxiety.

**Objective:**

The aims of this paper were to (1) describe the development and setup of a new internet-delivered acceptance and commitment therapy (iACT) program for patients with severe health anxiety using self-referral and a video-based assessment; and (2) examine the feasibility and potential clinical efficacy of iACT for severe health anxiety.

**Methods:**

Self-referred patients (N=15) with severe health anxiety were diagnostically assessed by a video-based interview. They received 7 sessions of clinician-supported iACT comprising self-help texts, video clips, audio files, and worksheets over 12 weeks. Self-report questionnaires were obtained at baseline, post-treatment, and at 3-month follow-up. The primary outcome was Whiteley-7 Index (WI-7) measuring health anxiety severity. Depressive symptoms, health-related quality of life (HRQoL), life satisfaction, and psychological flexibility were also assessed. A within-group design was employed. Means, standard deviations, and effect sizes using the standardized response mean (SRM) were estimated. Post-treatment interviews were conducted to evaluate the patient experience of the usability and acceptability of the treatment setup and program.

**Results:**

The self-referral and video-based assessments were well received. Most patients (12/15, 80%) completed the treatment, and only 1 (1/15, 7%) dropped out. Post-treatment (14/15, 93%) and 3-month follow-up (12/15, 80%) data were available for almost all patients. Paired t tests showed significant improvements on all outcome measures both at post-treatment and 3-month follow-up, except on one physical component subscale of HRQoL. Health anxiety symptoms decreased with 33.9 points at 3-month follow-up (95% CI 13.6-54.3, t_11_= 3.66, *P*=.004) with a large within-group effect size of 1.06 as measured by the SRM.

**Conclusions:**

Treatment adherence and potential efficacy suggest that iACT may be a feasible treatment for health anxiety. The uncontrolled design and small sample size of the study limited the robustness of the findings. Therefore, the findings should be replicated in a randomized controlled trial. Potentially, iACT may increase availability and accessibility of specialized treatment for health anxiety.

**Trial Registration:**

Danish Data Protection Agency, Central Denmark Region: 1-16-02-427-14; https://www.rm.dk/sundhed/faginfo/forskning/datatilsynet/ (Archived by Webcite at http://www.webcitation.org/6yDA7WovM)

## Introduction

Severe health anxiety is characterized by the preoccupation with fear of having a serious illness, which interferes with daily functions and persists despite medical reassurance [1]. In the Diagnostic and Statistical Manual of Mental Disorders, 4th edition (DSM-IV), the disorder was labeled hypochondriasis, but in the new Diagnostic and Statistical Manual of Mental Disorders, 5th edition (DSM-5) [2], severe health anxiety is now classified as illness anxiety disorder or somatic symptom disorder, if persistent and distressing physical symptoms are present. Severe health anxiety is highly prevalent in medical settings (0.3% to 8.5%) [1,3-5], and has an estimated lifetime prevalence of 5.7% in the general population [6].

Despite the high prevalence and the existence of effective psychological treatments [7], the disorder is still rarely diagnosed in the health care setting [8]. Health care professionals often lack the necessary knowledge about health anxiety, and when the disorder is recognized, there is often a lack of proper treatment options. Furthermore, a subgroup of patients tends to be care-avoidant, and this group is therefore at an even higher risk of diagnostic delay. As severe health anxiety is associated with extensive use of health care services [9,10] and occupational disability [11], and rarely remits if untreated [10], the limited accessibility to proper assessment and treatment is a major challenge to the health care system. Thus, evidence-based, easily accessible treatment options are warranted for this debilitating disorder.

Patient self-referral in combination with internet-delivered psychological treatment is a new approach that may facilitate treatment accessibility and broaden availability. As used here, internet-delivered treatment is a highly structured treatment that the patient accesses through a secure online platform. The patient is granted gradual access to content such as video clips, texts, audio files, and work sheets, and the treatment is guided and supported by a clinician through an embedded asynchronous message system. The basic principle is that internet-delivered treatment should target the same core treatment processes as face-to-face treatment, with only the mode of delivery changing [12]. internet-delivered treatment compares favorably with face-to-face treatment as it is unrestrained by geographical distance and interferes less with work and family obligations. Thus, it may inflict less strain on the patients' daily activities. Furthermore, studies suggest that internet-delivered treatment is less stigmatizing and therefore less of a barrier for seeking treatment than face-to-face treatment [13]. In addition, compared to treatment in an outpatient clinic, internet-delivered treatment may involve fewer societal costs due to absence from work and travel expenses.

internet-delivered treatment for common mental disorders has been investigated in well over 100 randomized trials, and a recent meta-analysis showed that internet-delivered cognitive-behavioral therapy (iCBT) for depressive and anxiety disorders can be just as effective as face-to-face-treatment [14]. In 3 randomized controlled trials (RCTs), Hedman-Lagerlöf and colleagues showed that iCBT can be highly effective for severe health anxiety [15-17]. Studies have also shown that the treatment effect is long-term and cost-effective [18].

Poor adherence to internet-delivered treatment is a widespread and well-documented concern [19,20], which may limit its potential effectiveness [21]. A recent meta-analysis found that the mean adherence to protocol was higher in internet-delivered acceptance and commitment therapy (iACT) compared to iCBT [22]. Acceptance and commitment therapy (ACT) is a new, promising treatment within the third wave of cognitive-behavioral therapies, which has shown a positive effect across a range of mental health conditions, including health anxiety [23-26]. ACT aims to increase “psychological flexibility” which is considered a core process across different disorders [27]. Patients with severe health anxiety report low psychological flexibility, which often narrows the behavioral responses to health anxiety [28]. Patients spend a lot of time and energy trying to control or avoid thoughts, feelings, and sensations associated with illness, limiting the focus on value-based activities and creation of a meaningful life. ACT deliberately does not focus on symptom reduction, since this focus can maintain the very same symptoms by continuing to control or avoid them. Instead ACT aims to improve 2 skills: acceptance of inner experiences and commitment to values-based behaviors. A recent trial on iACT for social anxiety and panic disorder suggested that iACT may be feasible for treating anxiety disorders [29]. However, to our knowledge, iACT for severe health anxiety has not previously been developed or tested.

The aims of this study were to (1) describe the development of a new treatment concept for patients with severe health anxiety encompassing patient self-referral, video-based diagnostic assessment, and treatment with iACT over 12 weeks; and (2) evaluate the feasibility and overall outcome of this new treatment concept. The feasibility evaluation included patients’ feedback provided both at the video-based assessment and post-treatment interview, self-reported questionnaires, and the patients’ attrition and adherence to the treatment. The evaluation did not apply a priori defined criteria of success, since this study was a proof of concept prior to conducting a larger, RCT.

## Methods

### Phase I: Development

Both the technical platform and the contents of the treatment were developed de novo (Multimedia Appendix 1). The treatment content was developed according to an evidence-based manual [30]. Decisions not informed by previous research were made in collaboration with an expert group consisting of 2 psychologists and 1 psychiatrist from the Research Clinic for Functional Disorders and Psychosomatics at Aarhus University Hospital in Denmark. This group was supplemented with 2 external psychologists with expertise in developing and conducting research on internet-delivered programs. Two focus group interviews with former patients were conducted to establish relevant themes to address in new treatment content (eg, patient videos). The technical platform was developed in collaboration with a Web-developer. New procedures—patient self-referral and video-based assessment—were applied.

#### Self-Referral and Video-Based Assessment

Self-referral was developed and tested as a new treatment entry through the clinic’s webpage, where patients could log into a portal using their unique Danish personal identification number. The self-referral procedure consisted of the following steps: (1) patients gave written consent for the clinician to access information on their health status through the electronic patient record prior to assessment, (2) patients were asked to describe their problem in an open text field “Please describe your health anxiety in your own words and how you feel at the moment,” and (3) patients completed baseline screening questionnaires measuring health anxiety symptoms among others. Video-based assessment was implemented to support nationwide recruitment. A shortened version of the semi-structured psychiatric interview, Schedules for Clinical Assessment in Neuropsychiatry (SCAN) [31], was adapted for this purpose in order to focus and shorten the assessment. The final version was screened for severe health anxiety and the corresponding diagnoses of hypochondriasis, illness anxiety disorder, and somatic symptom disorder. Furthermore, depression, anxiety disorders including obsessive-compulsive disorder and other somatoform disorders according to the International Classification of Diseases (ICD-10) were assessed.

#### Technical Platform

Accessibility, interactivity, contact, and technical solution are some of the focal points that define the functionality and appearance of internet-delivered treatment programs. The decisions regarding these focal points as they relate to the present study are described below.

##### Accessibility

Accessibility can range from full, open access to health care integrated programs with clinician-administered inclusion. Since this platform was developed and operated in a hospital setting, access was administered by health professionals to secure that the hospital’s legal responsibility was ensured. Treatment progress was monitored and access to treatment modules was granted consecutively by the clinician.

##### Interactivity

Interactivity can range from fixed content with no interaction to more responsive content such as online exercises or games that are adapted based on the patient’s interaction. The platform’s content was designed to be relatively fixed (text, audio files, video clips, illustrations), but included interactive work sheets which were completed by the individual patient using embedded text fields. All sheets were automatically collected and stored in a folder on the front page and shared with the patient’s personal clinician.

##### Contact

Contact can range from pure self-help to varying degrees and formats of contact (eg, written/telephone/video support to blended care where online tools are blended with face-to-face contact). The current platform enabled written, asynchronous clinician guidance (no real-time chat). This format was chosen to minimize interference with the patient’s daily activities. Guidance was provided throughout the 12 weeks of treatment. Every patient had a designated personal clinician and messages were answered within 48 hours on weekdays. The content of the written guidance was not predetermined or restricted, and no limit was established concerning contact frequency and length.

##### Technical Solution

Technical solution can range from tools designed for mobile apps to extensive Web-based programs. The current platform was developed as a Web-based app built upon Drupal, a flexible open source content management system (CMS) and development framework. This solution was chosen because the treatment material is comprehensive and specialized functionality had to be added to the platform. The following solutions were developed: (1) a 2-factor authentification login which interfaces with the Danish public login system (NemID); (2) an embedded, securely encrypted message system for written communication; (3) an automatic text message (short message system, SMS) notification system (eg, notifies patients about new content such as messages, modules, questionnaires); and (4) a clinician-monitored control panel with automatic notifications with information about patient activity (eg, new messages, work sheets, activity, or missed questionnaires). In addition, the responsive design allows access to the treatment program through mobile devices and tablets.

#### Treatment Content: Intervention

The treatment content was based on an empirically tested group-based ACT program for patients with severe health anxiety [24,30], and clinical experience with the manual and focus group interviews with former patients receiving group treatment. Literature on internet-delivered treatments was also reviewed. All treatment content aims to target and affect core structures of health anxiety such as experiential avoidance of unpleasant illness-related thoughts, feelings, and bodily sensations that inhibit values-based actions. The treatment program trains acceptance of these unpleasant inner states, clarifies values, and simultaneously promotes values-based exposure. The overall intention is to improve the patient’s psychological flexibility (ie, ensure they are able to experience a more flexible and value-based behavioral repertoire when health anxiety is present). This includes the distinction between what can and cannot be changed. Since ACT is a behavior therapy, the treatment works through both the processes of accepting what cannot be controlled (thoughts, feelings, sensations), and change what can be controlled (behaviors). The progression of treatment processes is illustrated in Table 1.

The number of modules in the original face-to-face manual was shortened from 10 to 7 (Textbox 1). Since this program was primarily based on self-help, the language was revised and simplified so that all content elements were self-explanatory. The front page features 10 widgets (messages, work sheets, acute help, and modules 1 to 7) (Multimedia Appendices 2 and 3). Each module has between 10 to 15 pages of content which is primarily text-based. The modules also contain audio files with guided exercises, such as mindfulness training, and patient videos with 5 former patients sharing their experience of health anxiety and treatment processes. The videos were produced to enhance treatment comprehension, recognition, and patient motivation. All videos and audio exercises are relatively short (between 4 to 20 minutes), and each module introduces at least 1 new video and audio exercise. Furthermore, illustrations were hand drawn, graphic design was added to structure different types of treatment elements, and written work sheets were embedded. All modules follow the same structure; the first page is an introduction to the following theme and a repetition of the previous one, next follows the main treatment content, and lastly a summary of the current module, clarifying questions and an introduction to weekly homework exercises. The homework consists of 2 alternating exercises each week: an audio exercise primarily based on mindfulness and a behavioral exercise based on values-based exposure (Multimedia Appendices 4-6).

### Phase II: Design of Feasibility Testing

#### Study Design and Setting

This feasibility study took place from April 2015 to October 2015 at the Research Clinic for Functional Disorders and Psychosomatics at Aarhus University Hospital in Denmark. It was an open trial with no control group. Self-report questionnaires were obtained at baseline prior to diagnostic assessment, at post-treatment, and at 3-month follow-up. Patients not completing any modules were excluded from the final analyses as the primary aim of the study was to evaluate the feasibility of the new treatment set-up and program. Treatment completion was defined a priori as 3 or more modules completed since the first 2 modules primarily consisted of psycho-education and did not explicitly include behavior change strategies. Semi-structured interviews with patients after end of treatment were conducted by the clinician assigned to the individual patient to explore the usability and acceptability of the procedure with self-referral, video-based assessment, as well as the treatment program. All participants provided written informed consent, and the study was approved by the Danish Data Protection Agency, Central Denmark Region (ID no. 1-16-02-427-14).

#### Participant Recruitment and Eligibility

A total of 15 patients were recruited, 10% of the projected sample size for the RCT. This was considered sufficient for the feasibility evaluation of the treatment concept including self-referral, assessment, and treatment. Patients were recruited through the clinic’s webpage, which announced information about the study, and presented a patient video about health anxiety. Patient self-referrals were screened for eligibility by a psychologist (first author) and then allocated to a video-based clinical assessment. The assessments were conducted by psychologists or psychiatrists trained in the use of the shortened version of SCAN [31]. Health information was assessed and the electronic patient record was examined before the interview. A psychiatrist provided supervision in regard to somatic questions. Eligible participants had to (1) report symptoms corresponding to fulfillment of the empirically-based diagnostic criteria for severe health anxiety in accordance with Fink et al [1]; (2) have a Whiteley Index-7 (WI-7) score above 21.4 (scale range 0 to 100), which is established as a clinically relevant cut-off score [10,24]; (3) be at least 18 years old; (4) be able to read and understand Danish; and (5) have a primary diagnosis of health anxiety if co-morbid disorders were present. Patients were excluded from the study if they (1) were at risk for suicide; (2) had current or previous episodes of psychosis; (3) had current abuse of alcohol, drugs, or medication; (4) were pregnant; and (5) did not give informed consent to the study.

**Table 1:** Treatment processes.

Module	Functional analysis	Mindfulness	Willingness and commitment	Values	Defusion	Acceptance
1	✓			✓		
2	✓	✓		✓		
3		✓	✓			✓
4		✓	✓	✓		
5		✓	✓	✓	✓	✓
6		✓		✓	✓	✓
7	✓					✓

**Overview of the treatment.**

Treatment targets for each module

**Module 1: The first important step**

- What are health anxiety and acceptance and commitment therapy?
- Overview and introduction to the treatment
- Motivation and values for participation

**Module 2: Your experiences managing health anxiety**

- Model of health anxiety and introduction to values
- Control, avoidance and creative hopelessness
- Mindfulness and rumination about illness

**Module 3: Challenging and accepting anxiety**

- Evolutionary function of bodily symptoms of anxiety
- The workability of control and avoidance behavior
- Challenging (exposure) and accepting anxiety

**Module 4: You get to know your values when taking steps**

- Values sorting
- Values compass
- Values-based exposure-hierarchy

**Module 5: What is stopping me?**

- Inner barriers (thoughts, feelings, bodily sensations)
- Defusion and acceptance
- Mindfulness and self-as-context

**Module 6: Self-compassion and boundaries**

- How stress affects bodily symptoms
- Balancing maladaptive “all-or-nothing” behavior
- Personal boundaries, open up and self-care

**Module 7: Relapse prevention**

- Repetition of treatment principles
- Consolidating personal insights from the treatment
- Normalization of relapse and relapse prevention

#### Measures

The mean sum score of the scales presented below were transformed into a 0 to 100 score point scale according to equation 1:


**(1) **Scale = [(score – minimum) ÷ (maximum – minimum)] x 100

This was chosen to facilitate the comparison of changes between measures in this and previous studies [24,32].

#### Primary Outcome

The WI-7 [33] is a 7-item measure of health anxiety symptoms including items such as “Do you often worry about the possibility that you have a serious illness?” Each item is scored on a 5-point Likert scale ranging from 0 to 4 (range 0 to 28), with higher scores indicating more severe health anxiety. WI-7 is broadly employed and has shown acceptable psychometric properties in primary care [10], and good sensitivity and specificity in screening for DSM-IV hypochondriasis [34].

#### Secondary Outcomes

The Symptom Checklist-92 (SCL-92) [35] subscale for depression (SCL-92 Dep) is a 13-item measure of depression (range 13 to 65). The subscale for anxiety (SCL-92 Anx) is a 10-item measure of anxiety (range 10 to 50). Each item is scored on a 5-point Likert scale ranging from 1 to 5, with higher scores indicating more severe illness.

The Short-Form Health Survey (SF-12) [36,37] with 12 items measures 2 dimensions of health-related quality of life (HRQoL): the physical component summary (PCS) and the mental component summary (MCS). Both scales range from 0 to 100, with higher scores indicating better physical and mental HRQoL.

Life satisfaction [38] is a 1-item measure scored on a 10-point Likert scale ranging from 0 to 10, where 0 and 10 encompass the “the worst possible life” and “the best possible life,” respectively.

The Acceptance and Action Questionnaire-II (AAQ-II) [39,40] is a 7-item measure of psychological flexibility. Each item is scored on a 7-point Likert scale ranging from 1 to 7 (range 10 to 70), with higher scores indicating better functioning.

#### Statistical Analysis

Data were summarized using means and standard deviations. The mean differences from baseline to post-treatment and from baseline to 3-month follow-up were analyzed using paired *t* tests. The assumptions behind the paired *t* test were assessed by graphical inspection of a quantile-quantile (Q-Q) plot of the differences and a Bland-Altman plot. An estimate of within group effect sizes was calculated using the standardized response mean (SRM) according to equation 2, by the formula, where X_1_ and X_2_ are the means of the measurements at time points 1 and 2, respectively. SD_DIFF_ is the standard deviation of the variable containing the difference between the 2 measurements.


**(2) **SRM = (X_1_ – X_2_) ÷ SD
_DIFF_

Adherence was measured as the mean number of modules completed, and exchanged messages in the program were summarized using means and standard deviations. All analyses were performed using Stata version 13 for Windows. The post-treatment interviews were conducted using semi-structured questions. The answers were not analyzed quantitatively.

## Results

### Patient Characteristics and Adherence

**Figure 1:**
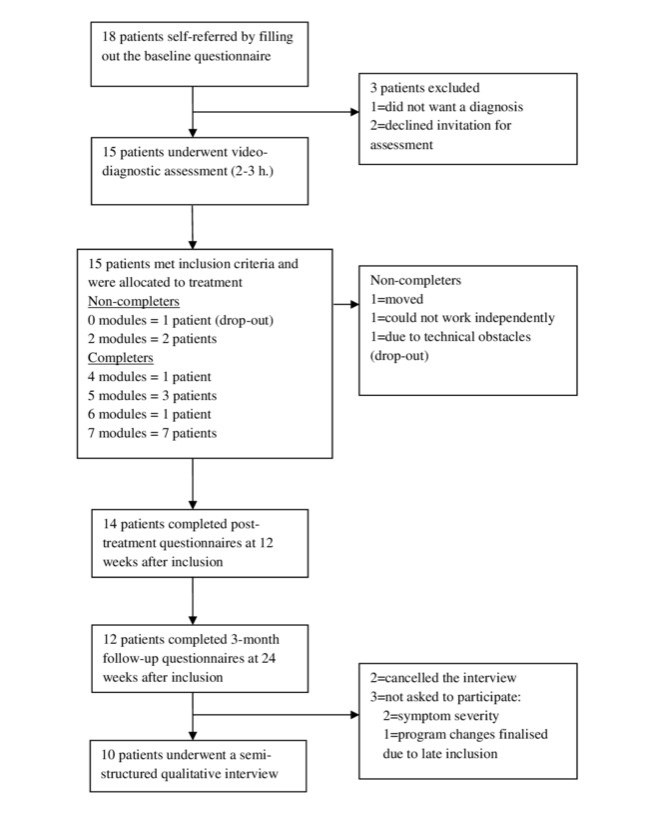
Study flowchart.

A total of 18 patients were self-referred of which 3 (3/18, 17%) withdrew prior to assessment (Figure 1). Of the patients, 15 (15/18, 83%), including 12 females (12/18, 67%) were assessed for eligibility, all of whom met the inclusion criteria and were included in the study. Socio-demographic and clinical details are shown in Table 2. Of the patients, 80% (12/15) completed treatment (ie, performed a minimum of 3 modules). The majority of the completers were considered active during all 12 weeks of treatment, independently of the number of modules finalized. One patient (1/15, 7%) dropped out prior to module 1 due to technical obstacles. Thus, post-treatment data on 14 (14/15, 93%) patients and 3-month follow-up data on 12 (12/15, 80%) patients were included in the final statistical analyses (Figure 1).

Of the patients, 12 (12/15, 80%) agreed to participate in the semi-structured interviews at post-treatment. Due to cancellations, only 10 interviews were conducted. The patient who dropped out and 1 of the non-completers participated in the interview ensuring a varied evaluation.

### Feasibility of Patient Recruitment and Assessment

Online patient recruitment through the clinic’s webpage attracted the patient population of interest. All self-referred patients were judged to have health anxiety as their primary problem based on the first screening and they were invited for assessment. Patients reported that it was easy to find the trial and go through the steps of self-referral.

At the end of the video-based interview, patients commented on their user experience. Overall, patients found it convenient, and the majority expressed feeling more secure at home than having to go to the hospital. A few patients felt anxious prior to the assessment due to the new video-format, and technical issues did interrupt a few interviews. In spite of this, most patients expressed that the video-format did not interfere negatively with their sense of contact with the clinician during the assessment. All clinicians had no prior experience with the video-format, and they reported positively about the amount of clinical information the assessment allowed for.

### Feasibility of the Treatment Platform and Content

The post-treatment patient interviews revealed overall satisfaction with relevance and progression of treatment themes and a positive response to the use of patient videos. There was no consistent pattern in content feedback (eg, some patients emphasized the importance of mindfulness exercises, and some patients did not use them at all). Patients sent an average of 12.9 (SD 7.76) messages (range 4-29) to their clinician, and clinicians sent an average of 18.5 (SD 3.46) messages (range 12-25) to their patient. Some patients expressed that this form of communication allowed for more honesty compared with earlier experiences with face-to-face therapy, whereas for others it was a hindrance. Therefore, some patients wrote many extensive messages where others consulted the clinician only to a limited extent. The clinicians reported that it was possible to have a good clinical sense of the patient. However, for those patients who did not use the clinician, the therapeutic reinforcement was missing to a larger extent. Furthermore, the patients were generally surprised by the amount of time they needed to complete a module.

### Changes in Outcome Measures

Mean, standard deviation, and percent changes at baseline, post-treatment, and 3-month follow-up are shown in the Table 3. Mean difference, CI, *P* values, and effect sizes are shown in Table 4. Patients reported statistically significant reductions in health anxiety symptoms from baseline to post-treatment with a difference in WI-7 score of 37.2 points (95% CI 24.1-50.4, *t*_13_= 6.11, *P*<.001), and at 3-month follow-up with a difference of 33.9 points (95% CI 13.6-54.3, *t*_11_= 3.66, *P*=.004) with large within-group effect sizes (SRM 1.06-1.63).

**Table 2:** Baseline characteristics of study sample (N=15).

Variable		Value
Age (years), mean (range)		38.8 (20.8-56.6)
**Sex, n (%)**		
	Female	12 (80)
	Male	3 (20)
**Education, n (%)**		
	Basic school (9-15 years)	9 (60)
	Further/higher education (<4 years)	6 (40)
**Work status, n (%)**		
	Full-time employed	7 (47)
	Part-time employed	3 (20)
	Unemployed	0 (0)
	Disability pension or flexible work	4 (27)
	Other (eg, maternity leave)	1 (7)
**Absence from work, n (%)**		
	No absence	10 (67)
	Full-time absence	3 (20)
	Part-time absence	2 (13)
**Living status, n (%)**		
	Alone	2 (13)
	With someone	13 (87)
Duration of health anxiety (years), mean (range)		15.2 (0.7-30)
Onset of health anxiety (years), mean (range)		23.6 (9-40)
**Psychiatric co-morbidity, n (%)**		
	Other anxiety disorders	4 (27)
	Depression	0 (0)
	Other somatoform disorders^a^	2 (13)

^a^Somatization disorder or undifferentiated somatoform disorder.

**Table 3:** Mean, standard deviation and percent change at baseline, post-treatment, and 3-month follow-up. WI-7: Whiteley-7 Index. SCL-92 Dep: symptom checklist-92 depression. SCL-92 Anx: symptom checklist-92 anxiety. PCS: physical component summary. SF-12: short-form health survey. MCS: mental component summary. AAQ: acceptance and action questionnaire.

Outcome measure^a^		Baseline (T1; N=15)	Post-treatment (T2; N=14)		3-month follow-up (T3; N=12)	
		Mean (SD)	Mean (SD)	%^b^	Mean (SD)	%^c^
**Primary outcome**						
	Health anxiety (WI-7)	80.9 (18.5)	43.6 (13.0)	–46	45.5 (29.6)	–44
**Secondary outcomes**						
	Depression (SCL-92 Dep)	40.4 (13.2)	21.2 (13.3)	–48	16.2 (13.6)	–60
	Anxiety (SCL-92 Anx)	43.8 (14.3)	27.5 (16.4)	–37	18.8 (12.6)	–57
	PCS (SF-12)	44.7 (15.8)	46.2 (11.8)	3	48.5 (13.8)	9
	MCS (SF-12)	28.7 (9.5)	43.6 (10.0)	52	44.3 (12.1)	54
	Life satisfaction	45.7 (12.2)	59.3 (18.2)	30	71.7 (14.0)	57
**Process measure**						
	Psychological flexibility (AAQ-II	37.9 (15.8)	61.4 (20.0)	62	69.6 (16.3)	84

^a^High score is many symptoms, except for life satisfaction, SF-12, and psychological flexibility. Scale 0 to 100.

^b^Percent change from baseline to post-treatment, measured by ((T1-T2)/T1) x 100.

^c^Percent change from baseline to 3-month follow-up, measured by ((T1-T3)/T1) x 100.

**Table 4:** Change in outcome measures and effect sizes. MD: mean difference. SRM: standardized response mean. WI-7: Whiteley-7 Index. SCL-92 Dep: symptom checklist-92 depression. SCL-92 Anx: symptom checklist-92 anxiety. PCS: physical component summary. SF-12: short-form health survey. MCS: mental component summary. AAQ: acceptance and action questionnaire.

Outcome measure		Baseline to post-treatment (N=14)				Baseline to 3-month follow-up (N=12)			
		MD	95% CI	*P* value	SRM	MD	95% CI	*P* value	SRM
**Primary outcome**									
	Health anxiety (WI-7)	37.2	24.1 to 50.4	<.001	1.63	33.9	13.6 to 54.3	.004	1.06
**Secondary outcomes**									
	Depression (SCL-92 Dep)	19.2	11.2 to 27.3	<.001	1.38	21.5	10.5 to 32.4	.001	1.24
	Anxiety (SCL-92 Anx)	16.3	3.3 to 29.2	.02	0.73	25.6	14.9 to 36.4	<.001	1.51
	PCS (SF-12)	–3.3	–11.9 to 5.3	.42	0.23	–5.1	–13.3 to 3.0	.19	0.42
	MCS (SF-12)	–15.0	–21.0 to –9.0	<.001	1.50	–15.3	–21.7 to –8.9	<.001	1.61
	Life satisfaction	–13.6	–23.9 to –3.3	.01	0.76	–27.5	–37.3 to –17.7	<.001	1.78
**Process measure**									
	Psychological flexibility (AAQ-II)	–23.5	–35.3 to –11.7	<.001	1.15	–31.2	–42.1 to –20.2	<.001	1.80

There were no significant changes in scores from post-treatment to 3-month follow-up which suggests stability of clinical outcome. Furthermore, patients reported significant reductions in symptoms of anxiety and depression, and significant increases in life satisfaction and psychological flexibility (Table 4). Effect sizes were moderate to large on all secondary outcomes (SRM 0.73-1.80), with the exception of the SF-12 PCS measuring physical functioning. Apart from life satisfaction, which further increased significantly from post-treatment to 3-month follow-up (SRM 0.96), there were no significant changes in scores from post-treatment to 3-month follow-up.

### Subsequent Revisions of the Treatment Program and Set-Up

The patient interviews gave rise to revision and further extension of some treatment themes and exercises. Furthermore, we learned that patients need to not only be motivated for internet-delivered treatment, but also to be able to work independently, prioritize their time with regard to treatment engagement, and have a minimum of technical know-how. To further improve adherence prior to the RCT, a patient recruitment video was developed to provide a clear picture of the treatment format, emphasizing the time and effort needed to participate in this type of treatment. This was also further addressed in the assessment interview. Patients who were ambivalent had up to 2 weeks to decide whether to enter the randomization for the following trial. Based on the experience with the drop-out of 1 patient due to technical obstacles, we included a question about computer skills in the baseline questionnaire. We also enquired more about this in the assessment interview. Finally, due to discussions raised about changes in medication during the pilot study, an extra inclusion criterion was added for the RCT to ensure that patients had been stable on anxiety medication for at least 2 months prior to inclusion.

## Discussion

### Principal Findings

The main finding of this pilot feasibility study was that iACT encompassing online self-referral, video-based assessment, and internet-delivered treatment was a feasible treatment concept for patients suffering from severe health anxiety. We found overall positive outcomes with large improvements in health anxiety symptoms and medium to large improvements in anxiety, depression, HRQoL, and life satisfaction, which improved further during the 3-month follow-up period. The observed reductions in health anxiety symptoms were similar to those reported in a previous, uncontrolled, face-to-face pilot study, and a subsequent RCT on group-delivered ACT for patients with health anxiety [24,32]. Since health anxiety is considered a chronic disorder if untreated [10], spontaneous remission is unlikely to have contributed significantly to this effect. It is often debated whether symptom measures should be used as a primary outcome in ACT trials, since the treatment does not focus on symptom reduction but instead aims to increase behavioral flexibility and valued living. In our study, we also measured psychological flexibility, which did show the largest improvement (SRM 1.80). This supports the model of change suggesting that increased psychological flexibility is associated with general psychological well-being and might even precede symptom reduction [28,41]. Thus, symptom reduction is a welcomed treatment outcome but should not be mistaken as a therapeutic goal in treatment. In the present study, only 1 (1/15, 7%) patient dropped out, and 12 patients (12/15, 80%) completed 12 weeks of treatment. This was comparable to iCBT for health anxiety which has shown a similar completion rate of 85% [15]. Since the majority of patients completed the treatment, iACT seems to be an acceptable and feasible treatment for severe health anxiety.

### Strengths and Limitations

The thorough standardized clinical assessment and well-defined diagnostic inclusion criteria were strengths of this pilot study. Moreover, both completers and non-completers participated in the post-treatment interview and most data were available for patients at follow-up. Lastly, the study employed validated clinical outcome measures. Even though this study did not employ a randomized design, most outcomes pointed in the same direction: symptoms improved following iACT. The most important limitation of this study was the uncontrolled study design and small sample size. The design was chosen based on the aims of this study, and the sample size did not limit the feasibility evaluation, which was the primary aim. The preliminary observed clinical changes should be replicated in a larger RCT to establish clinical relevance. Another limitation was that the video-based assessment was not evaluated systematically. We did discuss the technical and clinical implications at supervision, and asked the patients about their experience at the interview, but we did not collect our experiences in a structured manner. Lastly, the post-treatment patient interviews were not analyzed systematically and since they were conducted by the dedicated clinician, there was a risk of bias due to socially desirable answers.

### Patient Recruitment and Generalizability

Patient self-referral is rarely applied in a publicly funded health care system based on gatekeeping, where patients are referred primarily through the general practitioner. We found that the majority of those who self-referred fulfilled criteria for severe health anxiety, which may indicate that self-referral is a sensible recruitment procedure in patients with severe health anxiety. One explanation may be that patients with health anxiety often seek medical information via the internet, and may have come across the clinic’s webpage while searching for explanations for their symptoms. The patient video on the webpage was likely to have further increased recruitment sensitivity as many patients reported that they recognized their own health anxiety from the patient’s stories. Former studies support that patient self-referral is a feasible recruitment method for internet-delivered psychological treatment [42,43]. However, patient characteristics may differ compared to patients referred from the general practitioner to outpatient treatment. Prior to this study, we anticipated that the treatment setup would appeal to males and younger patients with less severe symptoms of health anxiety. Studies tend to find no age or gender difference in the distribution of health anxiety [10], yet males and younger patients seem to be less frequently referred to treatment [24]. However, in the present study, we recruited 80% (12,15) female patients, with an average age of 38.8 years. Moreover, patients had health anxiety for approximately 15 years and were quite impaired at referral, with an average WI-7 score of 81 (range 0 to 100). Online administration of questionnaires has shown to be a valid method for measuring symptoms of health anxiety, which suggests that the surprising level of symptom severity cannot be explained by this [44]. This might imply that a different patient subgroup was reached, even though the group did not differ in the expected direction. In the DSM-5, patients suffering from health anxiety are categorized as either care-seeking or care-avoiding [2]. It may be that self-referral appeals to more avoidant patients with health anxiety, thus making treatment accessible for a different subgroup. However, these speculations need to be investigated in a larger patient sample.

### Treatment Processes and Clinical Implications

The overall aim of iACT for health anxiety is to target and improve a patient's psychological flexibility, which previously has shown to be a central process of change [28,45]. This was done through several treatment processes as previously illustrated (Textbox 1). Specifically, we had a distinct focus on acceptance of inner experiences and values in order to broaden the behavioral repertoire from control and avoidance to values-based behaviors. This involved gradually changing behaviors from unworkable short-term strategies to long-term goals in line with the chosen values. The distinct focus on personal values in ACT compared to CBT may explain why iACT tends to have better adherence to protocol compared to iCBT [22]. Future studies should include process measures to further understand the role of values and investigate the mediational effect on symptoms.

Clinician guidance was added to this treatment program because studies generally find that unguided internet-delivered treatment has higher attrition [20,46]. The asynchronous message system enabled a more flexible and patient-tailored contact in terms of individual needs of guidance. This was also reflected in the wide range of messages exchanged between each patient and their allocated clinician. The aim of the written guidance was to motivate patients, to validate patient efforts, to clarify misunderstandings, and to personalize treatment content. This study did not measure the quality of therapeutic alliance, but a former review on internet-delivered treatment across patient populations found client-rated alliances close to those found in face-to-face studies [47]. However, the review also reported that clinicians rated the alliance lower than in face-to-face treatment. This is in line with the feedback from clinicians in the present study regarding patients who did not use their dedicated clinician. This may suggest that clinicians are affected more by the internet-delivered treatment modality than patients.

### Conclusion

Severe health anxiety is a disabling and costly disorder with a lack of accessible, specialized treatment options. Here, we showed that online self-referral, video-based assessment, and iACT may be a feasible treatment set-up for patients suffering from severe health anxiety. The promising outcome results also suggest potential efficacy for this iACT program which is currently being tested in a larger RCT. If proven effective, iACT may increase availability and accessibility of specialized treatment for health anxiety. Future studies need to evaluate the sensitivity of patient self-referral, and to compare characteristics in patients self-referred or referred through a health care professional, to determine whether different patient populations are reached.

## Appendix

Multimedia Appendix 1 Video demonstration of the program.

Multimedia Appendix 2 iACT program.

Multimedia Appendix 3 Graphic design elements aiming to structure the treatment content throughout the program.

Multimedia Appendix 4 Illustration of long term consequences of health anxiety.

Multimedia Appendix 5 Illustration of control and avoidance as a mean to escape unpleasant thoughts, feelings and bodily sensations; here illustrated as 3 monsters.

Multimedia Appendix 6 Illustration of the principle of graded exposure.

## Abbreviations

ACT: acceptance and commitment therapy
DSM-IV: Diagnostic and Statistical Manual of Mental Disorders, 4th edition
DSM-V: Diagnostic and Statistical Manual of Mental Disorders, 5th edition
HRQoL: health-related quality of life
iACT: internet-delivered acceptance and commitment therapy
iCBT: internet-delivered cognitive-behavioral therapy
MCS: mental component summary
PCS: physical component summary
RCT: randomized controlled trial
SCAN: Schedules for Clinical Assessment in Neuropsychiatry
SCL: symptom checklist
SF-12: short-form health survey
SRM: standardized response mean
WI-7: Whiteley-7 Index
