# Supplementary figures and images for: Development and Feasibility Testing of Internet-Delivered Acceptance and Commitment Therapy for Severe Health Anxiety: Pilot Study
Source: JMIR Ment Health. 2018 Apr 6;5(2):e28. doi: 10.2196/mental.9198 (PMC5938695; doi:10.2196/mental.9198)

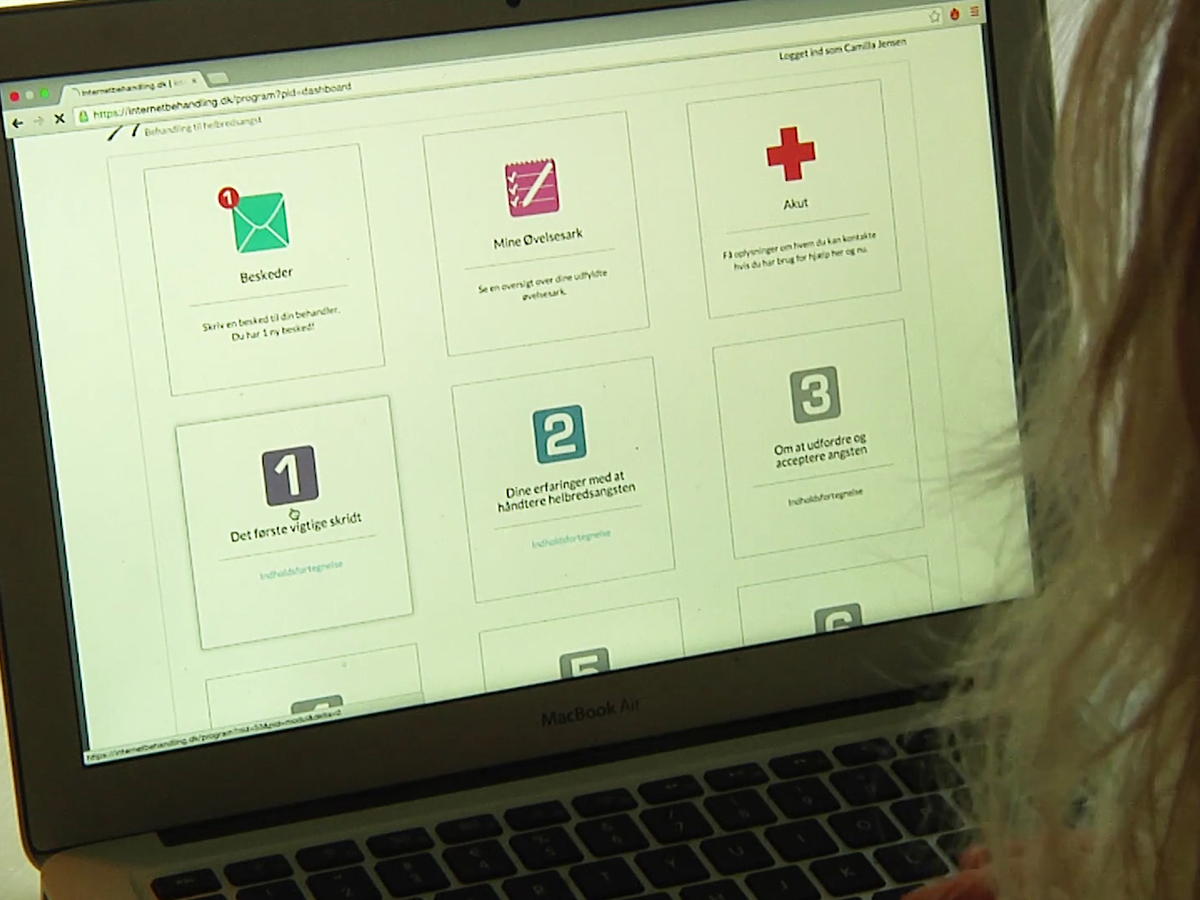

Supplement: Multimedia Appendix 2 [file mental_v5i2e28_app2.png]

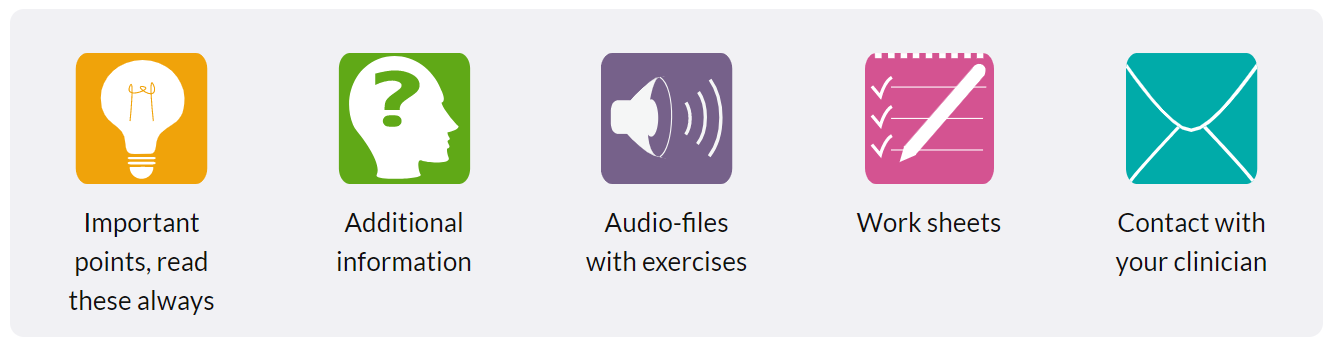

Supplement: Multimedia Appendix 3 [file mental_v5i2e28_app3.png]

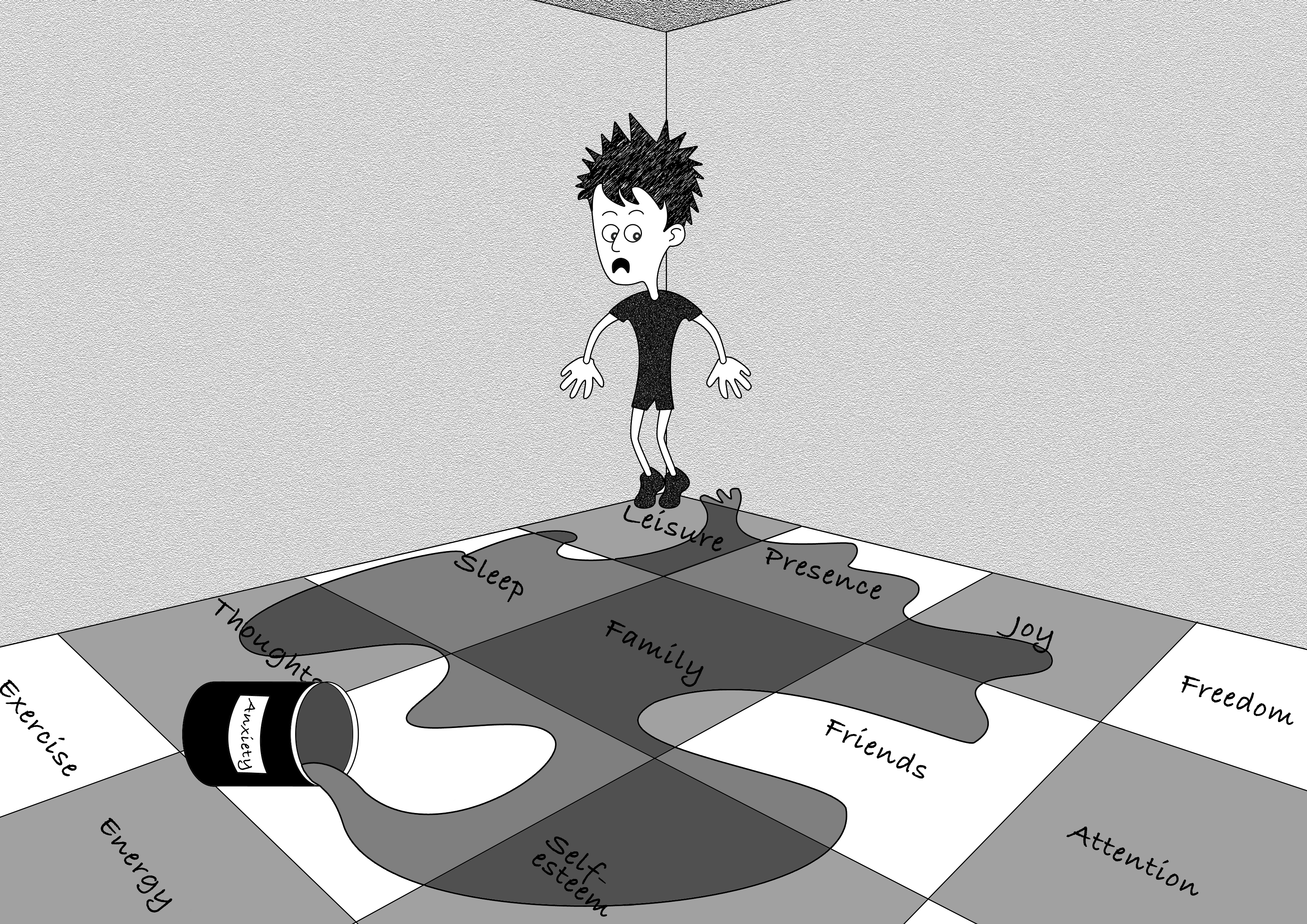

Supplement: Multimedia Appendix 4 [file mental_v5i2e28_app4.png]

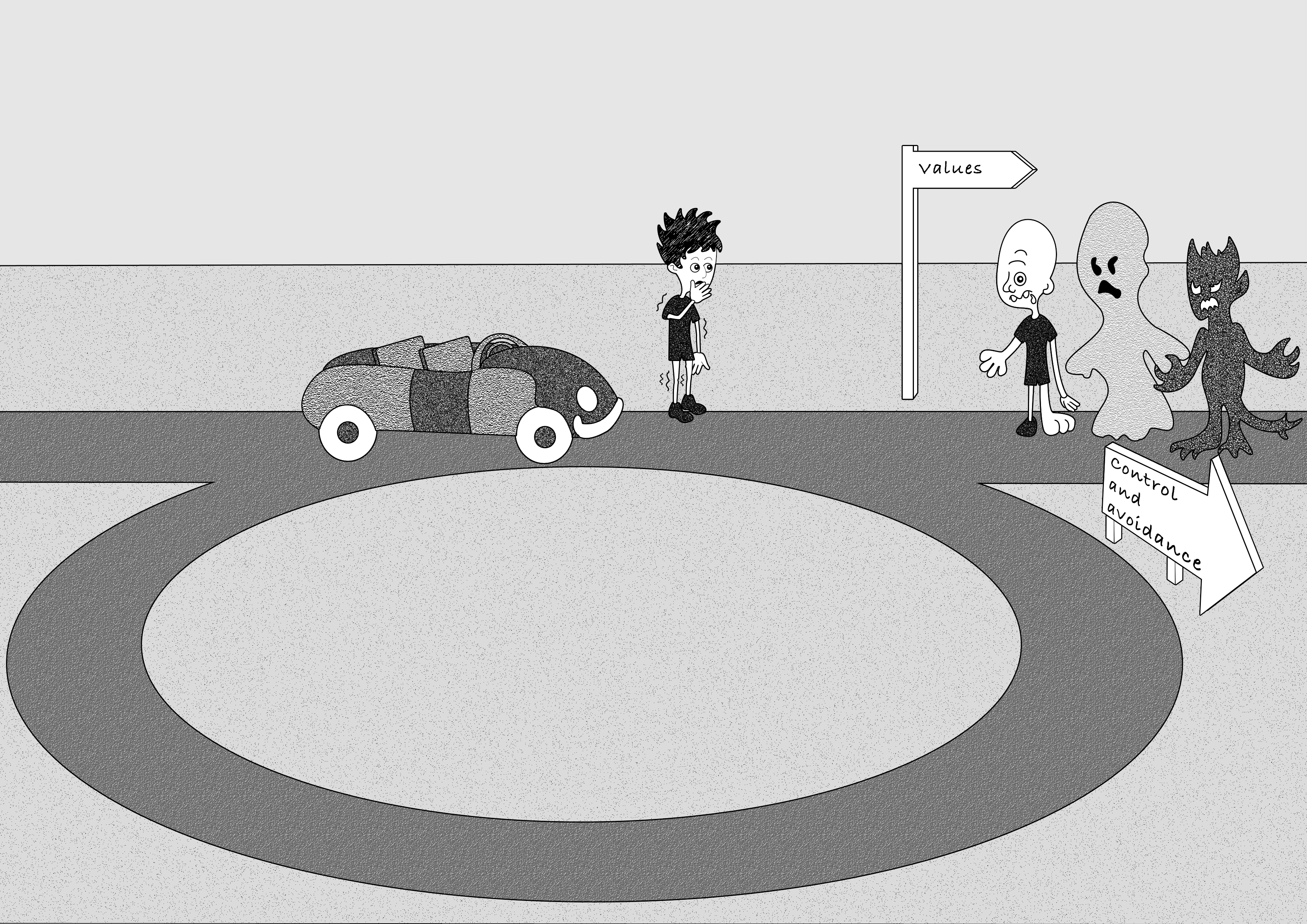

Supplement: Multimedia Appendix 5 [file mental_v5i2e28_app5.png]

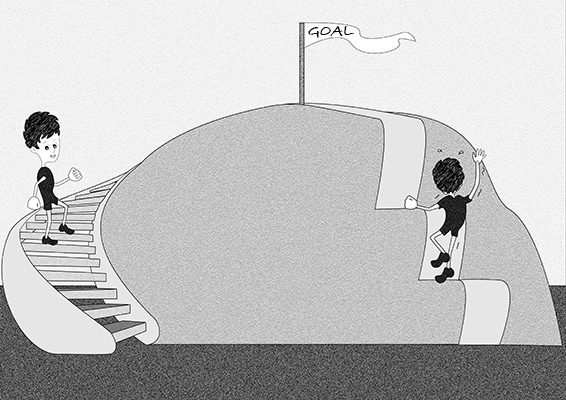

Supplement: Multimedia Appendix 6 [file mental_v5i2e28_app6.png]
